# Influence of modified starches on mental performance and physical activity following an exhaustive bout of exercise

**DOI:** 10.14814/phy2.15927

**Published:** 2024-02-04

**Authors:** Callie Herman, Gustavo Sandri Heidner, Laurel M. Wentz, Alexandra A. Shaver, Nicholas P. Murray

**Affiliations:** ^1^ School of Osteopathic Medicine Campbell University Lillington North Carolina USA; ^2^ Department of Exercise Science and Physical Education Montclair State University Montclair New Jersey USA; ^3^ Department of Nutrition and Healthcare Management Appalachian State University Boone North Carolina USA; ^4^ Department of Kinesiology University of Maryland College Park Maryland USA; ^5^ Department of Kinesiology East Carolina University Greenville North Carolina USA

**Keywords:** EEG, hydrothermally modified starches, maltodextrin, maximal exericse

## Abstract

Slow‐releasing carbohydrates may delay the effects of fatigue after exhaustive exercise. The purpose of this study was to observe the influence that hydrothermally modified starches (HMS) and traditional maltodextrin (MAL) supplements had on physical endurance and mental performance following exhaustive exercise. Male participants completed a VO_2_ max and 2 days of cycling sessions using a Velotron ergometer. Cycling sessions were performed at 70% of the VO_2_ max workload for 150 min. Supplements were consumed 30 min before cycling and during exercise at the 120‐min mark (1 g CHO/kg body weight). Brain activity was measured using a Neuroscan 64‐channel electroencephalogram cap. Go‐no‐go and N‐back tasks were performed before and after cycling bouts. Blood glucose, lactate, ketones, and urine‐specific gravity were measured before, during, and after cycling. VO_2_ and rate of perceived exertion were recorded in 15‐min intervals. Ketones increased significantly more for HMS than MAL from pre‐ to postcycling measurements (*p* < 0.05). Reaction times for go‐no‐go and N‐back were faster for HMS postexercise. Event‐related potential differences were present in both mental tasks following exhaustive exercise. HMS supplementation decreased the impact of cognitive and physical fatigue postexercise.

## 1 INTRODUCTION

A key to increasing endurance capacity is the ingestion of carbohydrate (CHO) supplement before and during endurance exercise. Consuming CHO provides a source of glucose for preventing hypoglycemia and aids in maintaining high CHO oxidation rates when glycogen stores in the muscles and liver begin to deplete (Mata et al., 2019; Roberts et al., 2011). Fast‐absorbing CHO's are traditionally used to facilitate these needs. However, they may inhibit fat mobilization and oxidation due to insulin stimulation (Baur et al., 2016; Roberts et al., 2011). It is hypothesized that a hydrothermally modified starches (HMS) that is digested and absorbed more slowly may have positive effects on metabolic and hormonal responses during exercise.

The key ingredient of HMS is superstarch, a CHO made from a GMO cornstarch (Roberts et al., 2011). It naturally has high levels of amylopectin. HMS has undergone a heat‐moisture treatment to alter its absorption rate into the bloodstream. The slower absorption rate causes a relatively low release of insulin into the bloodstream (Roberts et al., 2011). Previous studies have indicated that superstarches are more effective in treating and preventing hypoglycemia and may aid in sparing CHO stores by promoting the utilization of fats for energy during prolonged exercise (Baur et al., 2016; Roberts et al., 2011). It is expected that athletes will maintain more stable blood glucose when HMS is consumed compared to maltodextrins (MALs) due to subdued insulin response (Sands et al., 2008). Studies have also noted that ingesting a HMS before and during endurance cycling enhances fat oxidation and reduces CHO oxidation during exercise (Baur et al., 2016; Sands et al., 2008). Due to the sensitivity of lipolysis, small decreases in insulin are associated with increases in fat oxidation (Roberts et al., 2011). An increase in fat oxidation can preserve muscle and liver glycogen stores allowing for longer bouts of physical activity (Rodriguez et al., 2009). Glycogen stores are accessed during high‐intensity exercise because of the increased demand for glucose to maintain ideal performance. As muscle glycogen is depleted, athletes are susceptible to physical fatigue due to a lack of metabolites available for energy production (Rodriguez et al., 2009). Identifying the role glucose plays in athletic performance is important because it provides athletes with insight into how to buffer fatigue through nutrition. Discovering how and when glucose is used by the body in sports allows athletes and trainers to improve on current supplement recommendations to provide the brain with essential nutrients to promote stronger cognitive functioning in the later stages of exercise.

In recent years, considerable changes have occurred in understanding the role of carbohydrates during exercise. This has led to specific and more personalized recommendations on carbohydrate consumption during exercise (Jeukendrup, 2014). Carbohydrates are available to consumers in the form of bars, gels, and drink mixes. These forms allow individuals to easily ingest fuel before, during, and after bouts of exercise. Carbohydrates can be divided into the following three categories: rapid, resistant, and slow, based on digestibility (Sands et al., 2008). As the term implies, rapid‐digesting starches break down quickly, usually within 20 min of consumption. Resistant starches are the by‐products not absorbed by the small intestine after digestion. Slow‐digesting starches are digested in vitro between 20 and 120 min after ingestion (Sands et al., 2008).

Modified starches are ideal sources of glucose for extended periods because they have a low glycemic index, which has been shown to be effective in preventing hypoglycemia over time (Correia et al., 2008). The structural complexity of HMS contributes to its low glycemic index. HMS structures have extensive amylose and amylopectin, two polysaccharides that compose starches, branching that impedes amylase functioning (Baur et al., 2016). Amylase is the enzyme responsible for starch degradation. Because of this complexity, HMS digests at a slower rate, which reduces the rapid build‐up of glucose in the blood (Baur et al., 2016). Observing the metabolic impacts of HMS during prolonged exhaustive exercise may provide information on how to slow physical fatigue, maintain glucose concentrations, and improve mental performance.

Since carbohydrates are a popular fuel source in a variety of settings, it is important to continue investigating how new forms interact with the body. Traditional supplements (MAL) provide temporary relief during extended periods of exertion but have minimal long‐term benefits. Experiencing fatigue during sports or high‐stress occupations increases one's risk of injury due to the amount of effort required to execute desired actions. Cognitive impairments due to limited glucose cause athletes to respond slower and with less accuracy in fast‐paced environments, further increasing the risk of musculoskeletal injury (Murray & Rosenbloom, 2018). Evaluating the effectiveness of MAL and HMS during endurance exercise is critical for developing new techniques to minimize physical and mental fatigue over time.

Physical fatigue likely limits mental performance since glucose is the primary fuel for the brain and glucose concentrations decrease during prolonged endurance exercise without an exogenous source of carbohydrates (Murray & Rosenbloom, 2018). Event‐related potentials (ERP), specifically P300, are commonly used to examine the relationship between exercise and mental performance. P300 is indicative of neural activity necessary for basic cognition (Magnié et al., 2000; Pobric et al., 2022). With P300 serving as a measure of mental functioning, increases in P300 amplitude can be associated with the allocation of attentional demand. One factor that may elicit changes in P300 amplitudes is glucose availability. Considerable data is available indicating that hypoglycemia affects brain function (McCrimmon, 2021) as well as the impact of exercise on cognitive function (Audiffren et al., 2009; Chang et al., 2013; Lautenschlager et al., 2019). Since glucose is the brain's primary source of fuel, nutrition in sports may cause fluctuations in P300 amplitudes before and after exercise. Exhaustive exercise is one cause of hypoglycemia. If glucose is being used to combat physical fatigue, the brain must function with limited energy resources, ultimately weakening cognitive performance (Murray & Rosenbloom, 2018). Understanding the role of glucose in the body, particularly in skeletal muscle, is important because it can provide athletes with an explanation as to why their executive functioning may decrease as exercise duration increases.

The purpose of this study was to examine the influence that HMS and MAL supplements have on physical endurance and mental performance following exhaustive exercise. The first hypothesis was consuming the HMS supplement would improve cognitive performance following strenuous exercise in comparison to MAL supplementation. The second hypothesis was consumption of HMS would lead to a decrease in blood glucose and lactate when compared to MAL supplementation. Information gathered from this study can provide insight into effective supplementation methods for athletes and individuals with high‐stress occupations that require sharp mental performance during physically exhausting conditions.

## 2 METHODS

### 2.1 Participants

Ten male cyclists were recruited for voluntary participation. Qualified individuals had to have at least 1 year of competitive cycling experience, a BMI of less than 30 kg/m^2^, and be within 18–50 years of age. Of the 10 participants recruited, seven (age = 31 ± 10 years, mass = 78.6 ± 9.9 kg, VO_2_ max = 47.9 ± 8.7 mL/kg/min) completed all three testing sessions. Subjects completed a cardiovascular medical history questionnaire before participation. Participants were instructed to avoid ingesting any form of medication or supplement that enhanced performance, altered metabolism, or prevented high‐intensity exercise while being enrolled in the study. This study was approved by the East Carolina University Institutional Review Board for human subjects. Written consent was obtained from all participants before testing began.

### 2.2 Protocol

This study required 3 days of testing. The first testing session consisted of a peak oxygen consumption (VO_2_ max) assessment to determine the maximum workload. Following the VO_2_ max test, participants returned to the lab on two separate days for cycling sessions.

### 2.3 Peak oxygen consumption test

A Velotron Ergometer and Parvo metabolic cart (Parvo Medics, TrueOne 2400, Sandy, UT) were used to measure the participant's VO_2_ max. Age, height (m), weight (kg), and resting heart rate were recorded before the start of testing. Cycle seat and bar position measurements were documented and kept constant throughout each testing session. Skin fold measurements were collected on the first day of testing before exercise to ensure the fitness level of the participants; however, were not used in any analysis. A VO_2_ max protocol was programmed into the metabolic cart measuring gas exchange. At the start of testing, participants completed a 5‐min warm‐up phase at 70 watts. Once the warm‐up was complete, the metabolic cart signaled the ergometer to begin phase one. Two protocols were developed for participants within a weight range of 120–150 pounds and 150–180 pounds. The initial power levels were set to 100 and 190 watts, respectively. Wattage increased by 30 in 1‐min interval. The test was terminated when oxygen and carbon dioxide measurements plateaued, the participant could not maintain a cadence of at least 60 repetitions per minute, or when the participant signaled to stop cycling. During testing, heart rate and VO_2_ were recorded each minute, with rate of perceived exertion (RPE) recorded every 3 min. After the test was complete, participants completed a 3–5‐min cool‐down phase at 70 W.

### 2.4 Cycle testing

On each day of cycle testing, participants arrived at the lab having fasted for a minimum of 10 h and avoided strenuous exercise for 24 h before testing. Both cycling sessions followed the same protocol, with the only variation being in the type of supplement ingested (HMS/UCAN or MAL/Gatorade). Sessions were scheduled at least 7 days apart to allow participants time to recover from the exhaustive exercise.

### 2.5 Preexercise

When the participants arrived for testing, a urine sample was collected. Urine‐specific gravity (USG) was measured 30 min before exercise using an ATAGO Refractometer. Blood glucose, ketones, and lactate were also measured 30 min before exercise. Blood samples will be taken via finger prick. Blood glucose and ketone levels were measured with a Precision Xtra Blood Glucose & Ketone meter (Abbott Industries, Alameda, CA). Blood lactate levels were determined using a Lactate Plus meter. Once all resting biological markers had been measured, participants consumed HSM supplement which was the lemon‐lime flavor of UCAN electrolyte powder, low‐glycemic, complex carbohydrate or MAL supplement which lemon‐lime flavor of UCAN electrolyte powder, which is a low‐glycemic, complex carbohydrate or lemon‐lime flavor of Gatorade powder (1 g/kg bodyweight) dissolved in 1000 mL of chilled water within 30 min of the start of exercise. Both supplements came from a single lot so there was no variation in supplement ingredients or make. There was an additional examination of chemical composition other than what was reported by the manufacturers of the supplements. The order of supplements was randomly assigned so that there was no order effect on supplement type. The participants were required to drink the entire amount of fluid as quickly as they could. Once these measures were recorded, participants completed two mental performance tests.

### 2.6 Electroencephalogram

A SympAmps 2 Neuroscan 64‐channel electroencephalogram (EEG) system (Neuroscan Compumedics, Charlotte, NC) was used during the mental tasks to monitor electrical activity. The cap was centered on the participant's forehead at 10% of the distance between the nasion and inion. A conductive liquid was added to each Compumedics QuikCell in the 64 electrodes. Nuprep skin gel and alcohol swabs were used to exfoliate the areas above and below the eyes and the outer ear. Ear and eye (horizontal and vertical placement) electrodes were applied with a conductive gel. This process was repeated after the exercise for the second round of tasks.

### 2.7 Mental performance task

Go‐no‐go and N‐back tasks were utilized to assess mental performance. Task administration was controlled using Paradigm (www.paradigmexperiments.com/). Each task had four versions to avoid pattern memorization and were counterbalanced. For the go/no‐go task, participants watched pictures of car and bicycle parts and were instructed to only click the mouse key when bicycle parts appeared on the screen. All stimuli were presented for 150 ms and the intertrial interval was randomly set to a value between 1500 and 2500 ms. The N‐back task consisted of a series of letters and required subjects to click a mouse key each time the first letter of a set of four appeared on the screen. These tasks were performed before and after the 150‐min exercise session.

### 2.8 Exercise

USG, blood glucose, ketone, and lactate levels were reassessed using the same methods outlined in the preexercise phase. Participants cycled for 150 min at 70% of their VO_2_ max workload. During this time, heart rate, RPE, and VO_2_ readings were recorded in 15‐min intervals. VO_2_ was measured by placing the Parvo metabolic cart mouthpiece in the participant's mouth for approximately 2 min. A nose piece was added simultaneously to reduce the potential for air leaks while analyzing gas exchange. Based on VO_2_ and RPE, power was adjusted accordingly to maintain 70% of VO_2_ max workload.

At the 120‐min mark, participants consumed a reduced amount from preexercise of 0.7 g CHO/kg bodyweight ratio of the supplement dissolved in 700 mL of chilled water. Blood glucose, ketones, and lactate were assessed in 10‐min intervals beginning at the 130‐min mark until exercise cessation. Water consumption was recorded for the first day of cycling and matched during the second cycling session.

### 2.9 Recovery

Following the exercise, one final urine sample was collected to measure USG. Electrocardiogram readings were checked for accuracy before participants completed mental tasks. Blood glucose, ketones, and lactate were reassessed approximately 30 min after exercise. The EEG cap was prepared and placed on the participant prior to the performance tasks that occurred 30‐min postexercise. Each participant received a $50 gift card to compensate for their time.

### 2.10 Data processing

SPSS software (IBM, Version 26) was used to apply a generalized linear model to each biological marker and analyze the relationships between supplement type and time. EEG data were analyzed using MatLab and EEGLab software. Data were originally filtered at 1 Hz and 50 Hz, followed by artifact subspace reconstruction (ASR). To remove remaining artifacts and noise, data were processed with an 80 Hz filter, independent component analysis (ICA), and epochs were extracted from −0.3 to 5 s of stimuli onset. Only correct responses were included in calculations for go‐no‐go and N‐back mean response times.

### 2.11 Data analysis

USG was used to measure hydration and was not included in further analysis. Mean values for USG after HMS and MAL consumption did not significantly change before, at the start of, and after exercise. VO_2_ was monitored to measure workload. No significant changes for either supplement were observed (*p* = 0.05). To test the effects of glucose, lactate, and ketones, three 2 (Supplement Type) × 6 (Time) repeated measures ANOVA were conducted. Last, we conducted a 2 (Group) × 2 (Time) repeated measures ANOVA comparing RPE Time Start and RPE Time End.

## 3 RESULTS

### 3.1 Blood glucose

**FIGURE 1:**
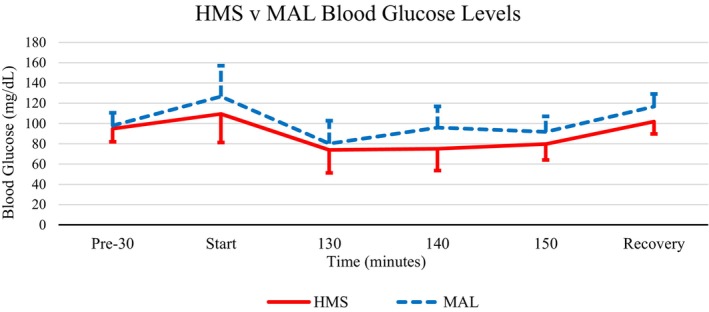
Mean blood glucose response to HMS (solid line) versus MAL (dashed line). Blood samples in the recovery phase were collected 30 min after exercise cessation. HMS, hydrothermally modified starches; MAL, maltodextrin.

For glucose, the analysis indicated a significant main effect for time (5, 65) = 11.703, *p* < 0.001, *η*_p_^2^ = 0.474. Glucose did not demonstrate a significant main effect for supplement type or supplement type × time (see Figure 1).

### 3.2 Blood lactate

**FIGURE 2:**
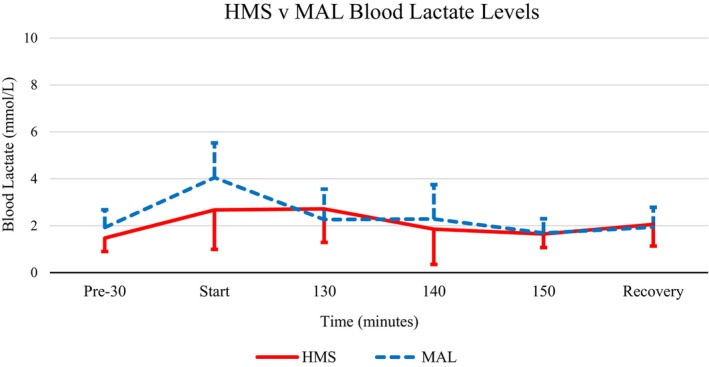
Mean blood lactate response to HMS (solid line) versus MAL (dashed line). Blood samples in the recovery phase were collected 30 min after exercise cessation. HMS, hydrothermally modified starches; MAL, maltodextrin.

Like glucose, lactate displayed a significant main effect for time (5, 65) = 4.260, *p* < 0.05, *η*_p_^2^ = 0.247 but did not demonstrate a significant main effect for supplement type or supplement type × time (see Figure 2).

### 3.3 Blood ketones

**FIGURE 3:**
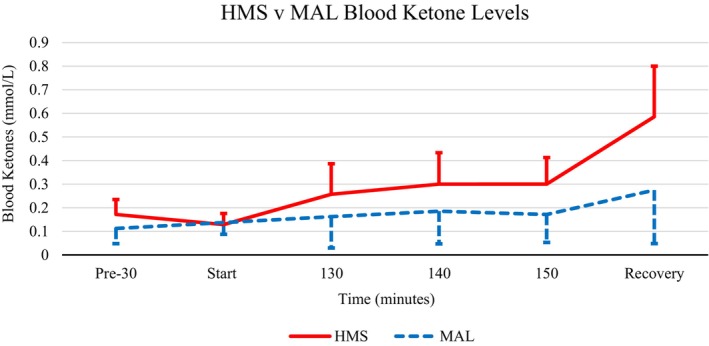
Mean blood ketone response to HMS (solid line) versus MAL (dashed line). Blood samples in the recovery phase were collected 30 min after exercise cessation. HMS, hydrothermally modified starches; MAL, maltodextrin.

The analysis indicated a main effect for supplement type (5, 55) = 7.933, *p* < 0.05, *η*_p_^2^ = 0.419, and a significant main effect for time (5, 65) = 20.007, *p* < 0.001, *η*_p_^2^ = 0.606, and a significant supplement type × time interaction (5) = 4.942, *p* < 0.001, *η*_p_^2^ = 0.275 (see Figure 3).

### 3.4 Go‐no‐go task

**FIGURE 4:**
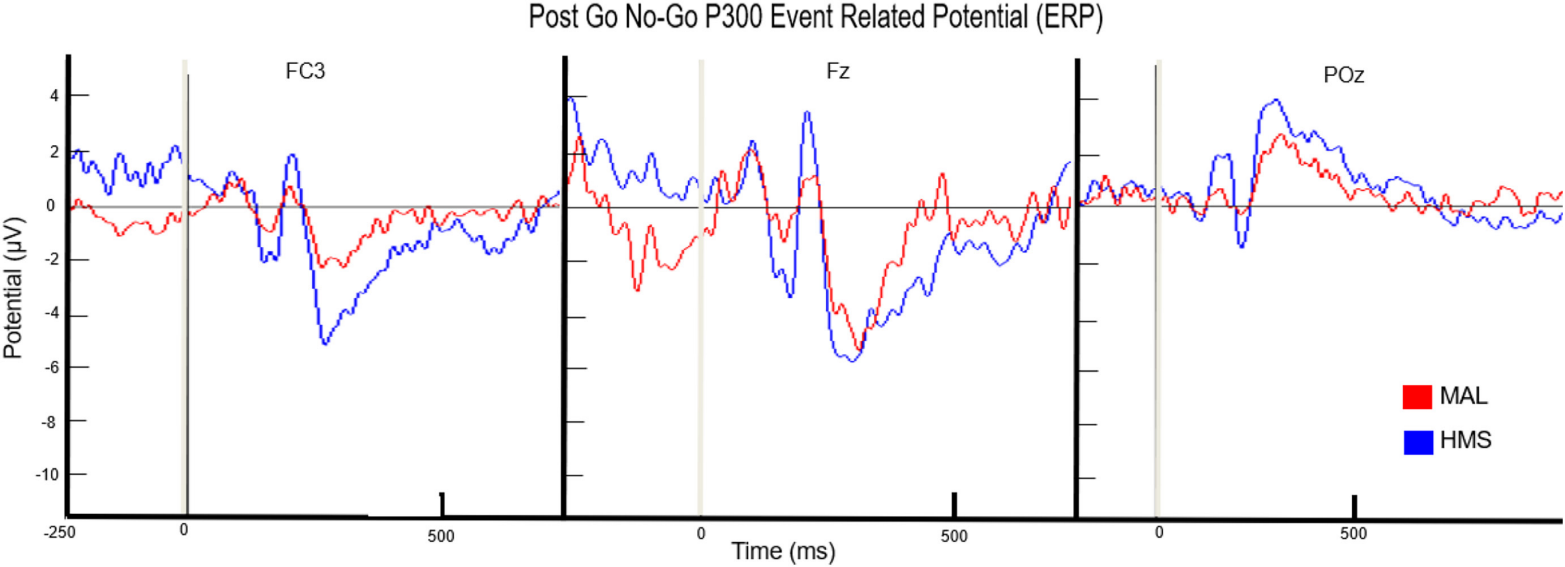
Post‐go‐no‐go P300 event‐related potential.

P3 amplitudes at frontal and parietal sites significantly decreased postexercise for both supplement types (*F*(1, 56) = 10.64, *p* < 0.01, *η*_p_^2^ = 0.32; *F*(1, 56) = 5.62, *p* < 0.05, *η*_p_^2^ = 0.22, respectively). There was a significant interaction between supplement type and time at frontal and parietal sites (*F*(1, 56) = 8.96, *p* < 0.01, *η*_p_^2^ = 0.31; *F*(1, 56) = 5.91, *p* < 0.05, *η*_p_^2^ = 0.18, *F*(1, 56) = 11.64, *p* < 0.01, *η*_p_^2^ = 0.34, respectively; see Figure 4).

**FIGURE 5:**
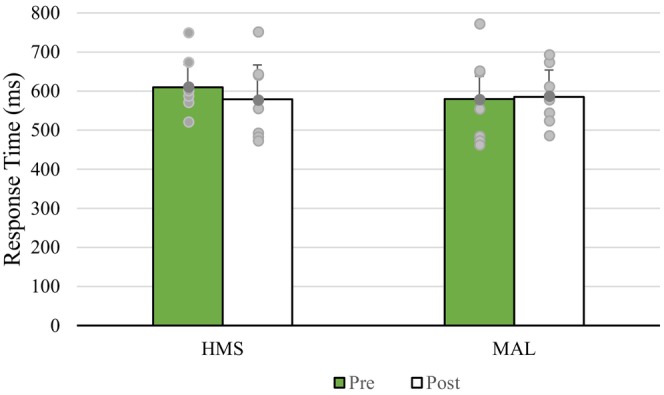
Mean response times for go‐no‐go tasks before and after exercise following HMS and MAL consumption. HMS, hydrothermally modified starches; MAL, maltodextrin.

In addition, post‐hoc tests for frontal and parietal sites demonstrated a significant decrease in amplitude at P3 for MAL in comparison to HMS after exercise. Response times for HMS decreased after cycling bouts (*F*(1, 6) = 6.52, *p* < 0.05, *η*_p_^2^ = 0.521) with no change for MAL postcycling (see Figure 5).

### 3.5 N‐back task

**FIGURE 6:**
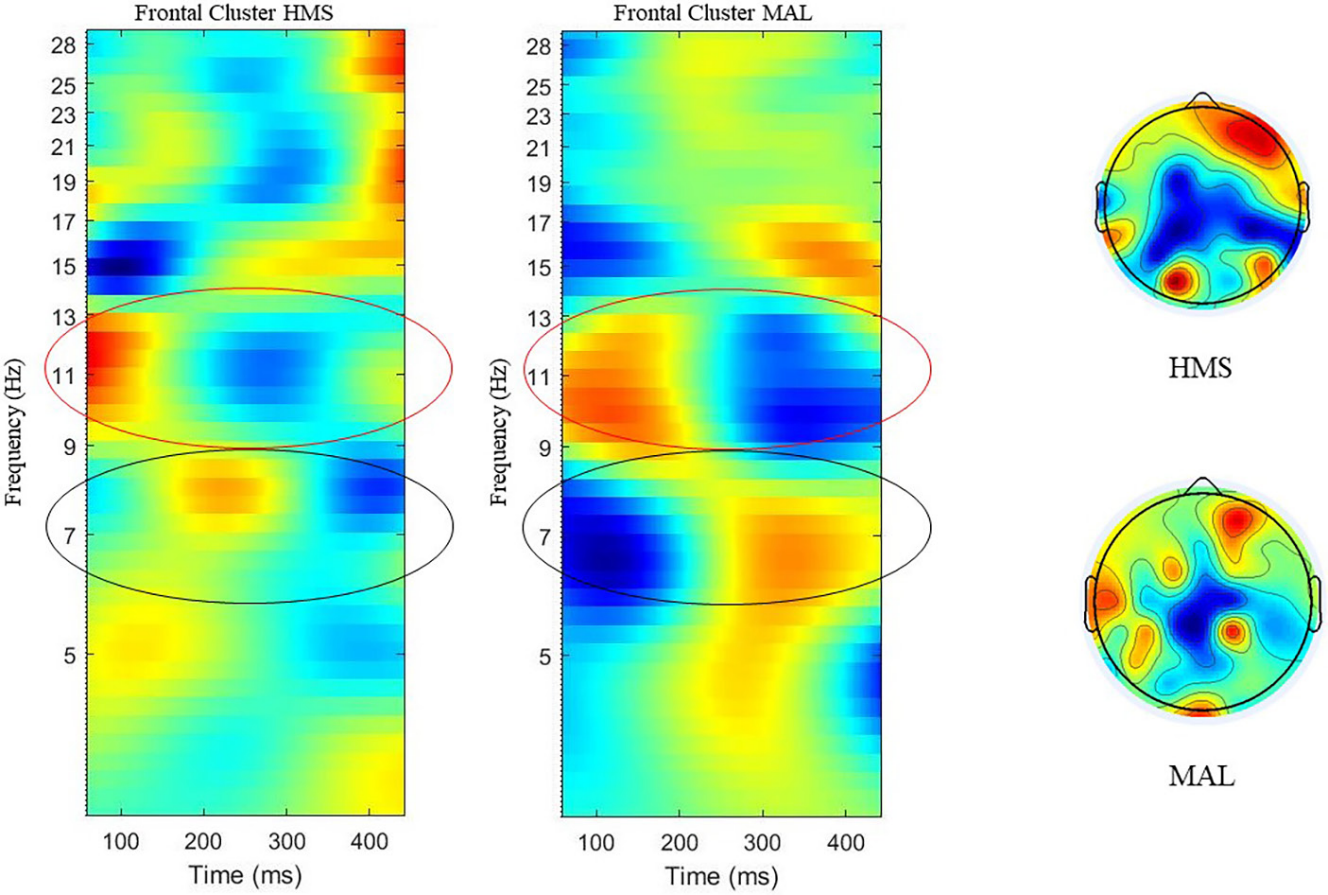
Post‐N‐back event‐related spectral perturbation.

Since the N‐back task is a measure of working memory, postexercise results were evaluated using an event‐related spectral analysis (ERSP). Results demonstrated a significant decrease in alpha and beta frequency band power (*p* < 0.05) at frontal and parietal electrodes (event‐related desynchronization, ERD) for HMS and a comparable increase in theta power (*p* < 0.05) at frontal electrodes (event‐related synchronization, ERS) for MAL (see Figure 6).

**FIGURE 7:**
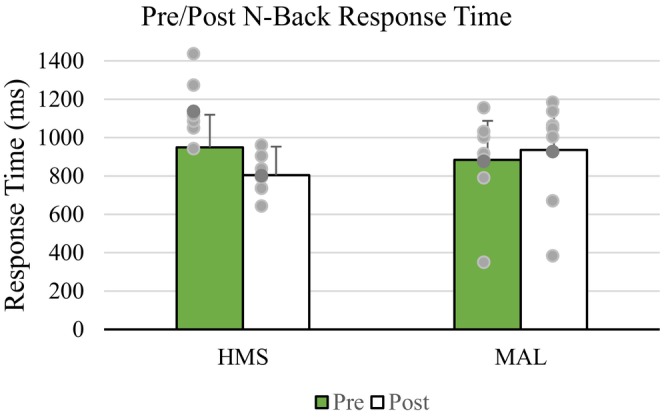
Mean response times for N‐back tasks before and after exercise following HMS and MAL consumption. HMS, hydrothermally modified starches; MAL, maltodextrin.

For MAL, high theta power (black circle) indicated an increase in activity within the central executive network. Low alpha power (red circle) for HMS reflected an increase in cortical inhibition and modifications of attentional demands. Response time decreased with HMS and increased with MAL consumption following exercise (see Figure 7). No significant effects over time were found with HMS (*p* = 0.05) but were observed in MAL (*p* < 0.05).

### 3.6 RPE

For RPE, the analysis indicated a significant main effect for time (1, 8) = 57.01, *p* < 0.001, *η*_p_^2^ = 0.8.77, and a significant interaction for supplement type × time, (1, 8) = 5.49, *p* < 0.05, *η*_p_^2^ = 0.407. RPE scores were significantly higher for MAL (Time 1: *M* = 10.2, SD = 2.48; Time End: *M* = 17.8, SD = 0.447) compared with HMS (Time 1: *M* = 9.6, SD = 2.19; Time End: *M* = 13.6, SD = 0.54).

## 4 DISCUSSION

This study compared how two carbohydrates with different molecular structures influence metabolic and cognitive processes in response to exhaustive exercise. We did see changes due to consuming HMS before and during exercise that influenced the physiological response to prolonged exercise; however, these changes did not differentiate serum glucose levels between HMS and MAL as expected. Typically, the stabilization would be due in part to the extensive amylopectin branching found in HMS structures (Sands et al., 2008). Complex amylopectin structures hinder the function of enzymes responsible for the breakdown of carbohydrates (Murray & Rosenbloom, 2018). Typically, enzyme impedance allows glucose to be released into the bloodstream at a slower rate and reduces the risk of hyperglycemia during exercise (Murray & Rosenbloom, 2018); however, this was not demonstrated here. Ingesting a high‐glycemic index food like MAL increases carbohydrate availability and leads to an upregulation of insulin (Chakrabarti et al., 2013).

Blood lactate concentration is one of the most common parameters measured during performance testing of athletes. In response to progressive exercise, lactate gradually increases at first and then more rapidly as exercise becomes more intense (Goodwin et al., 2007; Moreira‐Reis et al., 2022). In this experiment, lactate had a significant main effect over time. Although not significant, MAL lactate concentrations increased more during the pre‐exercise phase and at the start of exercise in comparison to HMS. Overall, there were larger variations although not significant in lactate measurements were observed with MAL consumption. Lactate build‐up during exercise has previously been viewed as a precursor for fatigue (Brooks, 2018).

Collectively, postexercise mental performance benefited from HMS consumption when compared to MAL. Results from the go‐no‐go task showed a reduction in P300 with MAL consumption. This reduction indicates cyclists experienced greater fatigue with MAL than HMS postexercise. Increases in P300 amplitude and decreases in P300 latency have been observed after moderate to intense aerobic exercise (Magnié et al., 2000). Depending on intensity and duration, exercise has been shown to influence cognitive performance by increasing or decreasing arousal. Thus, the greater the energy demand, the more attention is required to control movement (Brisswalter et al., 2002; Loprinzi et al., 2022). Greater fatigue and decreased mental performance with MAL may be due to lower ketones in the bloodstream. Typically, the brain primarily uses glucose as the primary fuel source of the brain; however, under semi‐fasted conditions, which was the case here, ketones may be the driver of brain energy (Jensen et al., 2020). Therefore, potentially the brain did not have the resources to manage attentional demand during mental tasks efficiently. The frontal lobe is responsible for executive functions like motor function and memory (Murray & Russoniello, 2012). This idea does need further examination in future research. Based on the N‐back ERSP, MAL required more frontal processing than HMS. Theta power activity reflects cognitive control functions in working memory. Higher theta power activity with MAL indicated that participants had to dedicate more effort to focus during N‐back tasks. Changes in alpha waves reflect the demands of working memory. Overall, there are a few limitations to this study including the use of meters to derive physiological variables. We did not collect blood to measure other variables such as insulin or nonesterified fatty acids, for example. In addition, we had another limitation was the inclusion of only males in our sample. This was due to the known differences in males and females on these variables. Given HMS led to lower alpha power, it can be inferred that this supplement reduced the demands on executive functioning by providing the brain with metabolites necessary for proper functioning (Rose & Richter, 2005; Sudo et al., 2017). In addition, RPE scores were significantly higher for MAL compared to HMS at the end of the exercise bout. Overall, this study has provided support for the claim that HMS supplements have the potential to stunt physical fatigue and improve mental performance for endurance athletes.

## AUTHOR CONTRIBUTIONS

HC, GSH, LW, and NPM conceived the study. CH, GSH, AS, and NPM collected the data. NPM and LW performed the primary data analysis. All authors were involved in the drafting and review of the manuscript.

## FUNDING INFORMATION

East Carolina University Research, Economic Development and Engagement Grant.

## ETHICS STATEMENT

The study was approved by the institutional review board from East Carolina University (Greenville, NC) and was conducted according to the Helsinki Declaration. All participants provided written informed consent.
